# Transcriptome analysis in hepatopancreases reveals the response of domesticated common carp to a high-temperature environment in the agricultural heritage rice–fish system

**DOI:** 10.3389/fphys.2023.1294729

**Published:** 2023-11-08

**Authors:** Xiangbing Cheng, Fangcheng Li, Gilbert Kumilamba, Jiayi Liao, Jiangwei Cao, Jiamin Sun, Qigen Liu

**Affiliations:** ^1^ Centre for Research on Environmental Ecology and Fish Nutrition of the Ministry of Agriculture, Shanghai Ocean University, Shanghai, China; ^2^ Key Laboratory of Integrated Rice-fish Farming, Ministry of Agriculture and Rural Affairs, Shanghai Ocean University, Shanghai, China; ^3^ Key Laboratory of Freshwater Aquatic Genetic Resources, Ministry of Agriculture and Rural Affairs, Shanghai Ocean University, Shanghai, China

**Keywords:** Qingtian paddy field carp, domestication, heat stress, RNA-seq, hepatopancreases

## Abstract

Qingtian paddy field carp (PF-carp) is a local carp cultivated in the paddy field of Qingtian, Zhejiang. This rice–fish co-culture system has been recognized as one of the Globally Important Agriculture Heritage Systems (GIAHS). PF-carp has been acclimatized to the high-temperature environment of shallow paddy fields after several centuries of domestication. To reveal the physiological and molecular regulatory mechanisms of PF-carp, we chose to use 28°C as the control group and 34°C as the treatment group. We measured biochemical parameters in their serum and hepatopancreases and also performed transcriptome sequencing analysis. Compared with the control group, the serum levels of malondialdehyde (MDA), glucose (GLU), glutathione peroxidase (GSH-Px), catalase (CAT), alanine aminotransferase (ALT), and aspartate aminotransferase (AST) show no significant change. In addition, superoxide dismutase (SOD), GSH-Px, and CAT also show no significant change in hepatopancreases. We identified 1,253 differentially expressed genes (DEGs), and their pathway analysis revealed that heat stress affected AMPK signaling pathway, protein export, and other biological processes. It is worth noting that protein processing in the endoplasmic reticulum (ER) was the most significantly enriched pathway identified by the Kyoto Encyclopedia of Genes and Genomes (KEGG) and gene set enrichment analysis (GSEA). Significantly higher levels of HSP40, HSP70, HSP90, and other ubiquitin ligase-related genes were upregulated. In summary, heat stress did not lead to tissue damage, inflammation, oxidative stress, and ER stress in the hepatopancreases of PF-carp. This study provides valuable insights into the adaptation mechanism of this species to the high-temperature environment of paddy fields.

## 1 Introduction

Modern human civilization has developed based on the successful domestication of various plants and animals (Diamond, 2002). The earliest truly domesticated fish is the common carp (*Cyprinus carpio*) (Balon, 2004). Influenced by geographic, cultural, and other factors, the common carp has been domesticated into a variety of local varieties that are widely distributed in different farming systems (ponds or paddy fields). Due to the disparity in altitude and the few plains in Qingtian, Zhejiang, humans have domesticated the most successful paddy-farmed fish in this region and have been named Qingtian paddy field carp (PF-carp). In Qingtian, a system of rice–fish symbiosis has been created by the integration of PF-carp and rice farming. As one of the first Globally Important Agricultural Heritage Systems (GIAHS), this system was acknowledged by the Food and Agriculture Organization (FAO) in 2005 (Lu and Li, 2006).

From rivers and lakes to paddy fields, the environment in which common carp live has changed dramatically. Common carp is a natural demersal fish inhabiting in the lower layer of rivers and lakes, where the temperature is more stable and less susceptible to external environmental influences. However, paddy fields have a small water body and are shallow (5–25 cm) compared to rivers and lakes (Chen, X. et al., 2021). The shallow paddy water environment is often hot in the summer afternoons due to the intense sunlight. According to our monitoring, the summer temperature of the paddy field where PF-carp lives is approximately 34°C. Yet, PF-carp has been domesticated in this environment for more than 12 centuries (Xie et al., 2011). Therefore, we hypothesize that they have been domesticated with special physiological and molecular regulatory mechanisms to adapt to the high-temperature environment of shallow paddy fields in Qingtian.

In order to investigate the adaptation mechanism of this species to the high-temperature environment of shallow paddy fields, we measured its biochemical parameters and performed RNA-seq analyses. The main objectives of our study were to characterize 1) the physiological changes in PF-carp in response to the high-temperature environment of shallow paddy fields and 2) the major signaling pathways and genes involved in the adaptation of PF-carp to the high-temperature environment of shallow paddy fields.

## 2 Materials and methods

### 2.1 Ethical statement

The experiments were conducted in accordance with the Guidelines for the Care and Use of Laboratory Animals in China. The animals used in this study were cultured and euthanized following the terms approved by the Institutional Animal Care and Use Committee at Shanghai Ocean University (Shanghai, China) with approval number: SHOU-DW-2018-026.

### 2.2 Animals

Fifty-four healthy PF-carp juveniles (weighing 104.69 ± 3.08 g and measuring 14.65 ± 0.46 cm in length) used in this experiment were procured from Yugong Ecological Agricultural Technology Co., Ltd (Qingtian, Lishui, Zhejiang, China). They were then transported to the PF-Carp Research Center (Qingtian, Lishui, Zhejiang, China) for a 7-day acclimation period. They were randomly divided into three circular tanks (18 fish per tank). Furthermore, they were acclimatized in laboratory settings with the aerating water maintained at 28°C ± 0.5°C and dissolved oxygen levels of approximately 7 mg/L. The water was changed daily, and the fish were given artificial food twice daily.

### 2.3 Experimental design and sample collection

We randomly selected nine individuals in three tanks after 7°days of acclimatization to serve as the control group. Then, the other experimental fish were elevated from 28°C to 34°C at a rate of 1°C per hour and maintained at 34°C for 24 h.

After maintaining the experimental temperature for 0, 2, 6, 12, and 24 h, samples were collected at each time point. Three PF-carp were randomly selected from each of the three tanks and anesthetized with MS-222 (300 mg/L) prior to sampling. A syringe was used to draw blood from the caudal vessel. Then, the fish were immediately dissected, and their hepatopancreases were collected for examination. The obtained samples were immediately frozen in liquid nitrogen and stored at −80 C until subsequent use. The same sampling procedure was applied to the control group.

### 2.4 Biochemical parameter determination

The changes in superoxide dismutase (SOD), malondialdehyde (MDA), glutathione peroxidase (GSH-Px), catalase (CAT), glucose (GLU), triglyceride (TG), alanine aminotransferase (ALT), and aspartate aminotransferase (AST) levels in the serum were determined. The supernatants of hepatopancreatic tissue homogenate were used for oxidative stress analysis, including SOD, MDA, GSH-Px, and CAT. According to the standard protocols, all the biochemical parameters were determined using reagent kits (Jiancheng Institute of Biotechnology, Nanjing, China).

### 2.5 Transcriptome sequencing

In this study, we selected hepatopancreatic tissues subjected to 6 h of heat stress for RNA-seq as the GM group. The TRIzol reagent (Invitrogen, Carlsbad, CA, United States) was used to isolate RNA of hepatopancreases (*n* = 3 per group); genomic RNA was removed using RNase I (Takara, Shanghai, China). Bioanalyzer 2100 (Agilent Technologies, United States) was used to determine RNA quality, and then ND-2000 (NanoDrop Technologies, United States) was used to quantify RNA. OD 260/280 ≥ 1.8 and OD 260/230 ≥ 1 were used for sequencing libraries.

These RNAs were reversed into cDNAs after a quality control process, and sequencing was carried out by Major Bioinformatics Technology Co., Ltd. (Shanghai, China) on Illumina NovaSeq 6000 (Illumina, United States). All transcriptome datasets can be found in the Sequence Read Archive (SRA) of the National Center for Biotechnology Information (NCBI). The accession number of the GM group was PRJNA1002641. RNA-seq results from a concurrent experiment were used as a control group (GC group), and its accession number was PRJNA971384.

### 2.6 Transcriptome analysis

SeqPrep and Sickle software programs were used to eliminate low-quality raw reads, and then the clean reads were mapped to the genome of common carp (accession number: ERS541549) (Xu et al., 2014) using HISAT 2 software and then aligned using StringTie. The levels of gene expression were estimated using transcripts per million reads (FPKM) in the present study. Genes with |log_2_FoldChange| ≥ 2 and p-adjust <0.05 were regarded as differentially expressed genes (DEGs) using DESeq2. Furthermore, GOATOOLS software and R package were used to perform GO and KEGG enrichment analyses, respectively. The gene sets of the KEGG pathway were used to perform further GSEA using GSEA 3.0 software.

### 2.7 Real-time quantitative PCR validation

We randomly chose nine DEGs for real-time quantitative PCR (RT-qPCR) verification to assess the accuracy of the transcriptome sequencing results. Primer Premier v.6.0 was used to design primers for DEGs (Supplementary Table S1). RT-qPCR was performed using the ChamQ SYBR Color qPCR Master Mix (2X) (Novozymes Bio, Nanjing, China) on a real-time fluorescence quantitative PCR instrument (ABI 7300, United States). The PCR conditions were as follows: 95°C for 5 min, followed by 40 cycles of 95°C for 5 s, 55°C for 30 s, and 72°C for 40 s. Gene expression levels were standardized relative to *β-actin* and calculated using Ct values (2^−ΔΔCT^).

### 2.8 Statistical analysis

All statistical analyses were carried out using SPSS 19.0. All results were analyzed using one-way ANOVA. Values are presented as mean ± standard deviation (mean ± SD). *p* < 0.05 was considered significant. In addition, GraphPad Prism 9 was used to display the results.

## 3 Results

### 3.1 Changes in biochemical indicators after heat stress

When PF-carp was maintained at 34°C, significant differences in the activity of SOD in serum (*p* < 0.05)were observed between the control and treatment groups at 6 h and 12 h. For the TG content in serum, significant changes were also observed to be higher in the treatment group than the control group at 12 h and 24 h (*p* < 0.05). However, there were no significant changes in the contents of MDA and GLU and the activities of GSH-Px, CAT, ALT, and AST in serum (*p* > 0.05) (Tables 1, 2).

**TABLE 1:** Effects of the oxidative index of the enzymatic type in the serum of PF-carp during heat stress.

Group	SOD activity (U/mL)	MDA content (nmol/mL)	GSH-Px activity (U/mL)	CAT activity (U/mL)
G	291.27 ± 16.74^c^	6.91 ± 0.31	187.95 ± 8.08^abc^	1.03 ± 0.18^cd^
G0	304.12 ± 21.81^bc^	7.99 ± 0.50	185.65 ± 18.87^abc^	1.26 ± 0.25^c^
G2	309.29 ± 23.43^bc^	7.97 ± 0.29	191.72 ± 17.04^abc^	1.42 ± 0.01^abc^
G6	341.51 ± 4.49^ab^	7.90 ± 0.22	170.50 ± 12.54^bcd^	0.63 ± 0.08^d^
G12	357.85 ± 7.95^a^	8.18 ± 0.33	183.77 ± 9.79^abc^	0.65 ± 0.03^d^
G24	313.66 ± 13.91^bc^	8.10 ± 0.24	182.05 ± 10.00^abc^	0.64 ± 0.19^d^

The values presented are the sum of the means and standard deviations (mean ± SD) of three replicates. Values in the same column with different lowercase letters indicate significant differences (*p* < 0.05). G, G0, G2, G6, G12, and G24 represent the control group and heat stress at 0, 2, 6, 12, and 24 h, respectively.

**TABLE 2:** Effects of metabolism and hepatopancreatic injury in the serum of PF-carp during heat stress.

Group	GLU content (mmol/L)	TG content (mmol/L)	ALT activity (U/L)	AST activity (U/L)
G	6.08 ± 0.50	2.57 ± 0.34^c^	1.30 ± 0.29^bcde^	7.78 ± 1.18^ab^
G0	9.93 ± 1.65	2.96 ± 0.17^bc^	1.58 ± 0.43^abcd^	9.04 ± 0.54^ab^
G2	9.73 ± 1.24	2.81 ± 0.35^bc^	1.83 ± 0.40^abc^	8.46 ± 1.45^ab^
G6	7.21 ± 1.13	3.15 ± 0.42^bc^	1.91 ± 0.35^ab^	10.65 ± 0.65^a^
G12	6.91 ± 0.59	3.62 ± 0.35^ab^	1.79 ± 0.09^abc^	9.28 ± 1.48^ab^
G24	7.66 ± 0.60	3.70 ± 0.55^ab^	1.72 ± 0.35^abcd^	9.89 ± 0.95^ab^

The values presented are the sum of means and standard deviations (mean ± SD) of three replicates. Values in the same column with different lowercase letters indicate significant differences (*p* < 0.05). G, G0, G2, G6, G12, and G24 represent the control group and heat stress at 0, 2, 6, 12, and 24 h, respectively.

The MDA content in hepatopancreases of the treatment group was significantly higher than that of the control group at 24 h (*p* < 0.05). In contrast, the activities of SOD, GSH-Px, and CAT were not significantly changed (*p* > 0.05) (Table 3).

**TABLE 3:** Effects of the oxidative index of the enzymatic type in the hepatopancreas of PF-carp during heat stress.

Group	SOD activity (U/mgprot)	MDA content (nmol/mgprot)	GSH-Px activity (U/mgprot)	CAT activity (U/mgprot)
G	535.64 ± 22.04^abcd^	0.69 ± 0.11^b^	252.32 ± 28.49	19.06 ± 2.79^a^
G0	576.52 ± 60.53^abc^	0.88 ± 0.09^ab^	222.95 ± 15.90	19.45 ± 2.76^a^
G2	587.90 ± 26.17^ab^	0.97 ± 0.11^ab^	228.94 ± 15.77	19.86 ± 1.65^a^
G6	520.76 ± 3.72^bcd^	0.81 ± 0.04^ab^	225.89 ± 29.99	14.98 ± 2.30^ab^
G12	520.76 ± 35.25^bcd^	0.88 ± 0.09^ab^	263.16 ± 27.71	15.71 ± 2.45^ab^
G24	568.41 ± 21.71^abc^	1.21 ± 0.11^a^	269.97 ± 24.70	19.83 ± 3.27^a^

The values presented are the sum of means and standard deviations (mean ± SD) of three replicates. Values in the same column with different lowercase letters indicate significant differences (*p* < 0.05). G, G0, G2, G6, G12, and G24 represent the control group and heat stress at 0, 2, 6, 12, and 24 h, respectively.

### 3.2 Overview of RNA-seq

We used six samples for transcriptome sequencing with three replicates for the hepatopancreatic tissue in the two distinct groups. After filtering low-quality reads, 272,933,850 clean reads were obtained. Parameter statistics of clean reads among two groups were Q20: 97.84%–98.21%; Q30: 93.88%–94.62%; GC content: 48.4%–50.43%; and error rate: 0.0246%–0.0254%. The mapping of clean reads to the reference genome was also carried out, and the total mapped ratio was in the range of 78.07%–79.87% (Table 4).

**TABLE 4:** RNA-seq library sequencing data statistics.

Sample	Raw reads	Clean reads	Q20 (%)	Q30 (%)	GC content (%)	Error rate (%)	Total mapped rate (%)
Control group (28°C)							
GC1	55,559,072	49,347,690	97.84	94.01	48.84	0.0254	79.57
GC2	45,756,400	44,193,878	98.03	94.11	48.8	0.0251	79.71
GC3	43,748,700	42,303,354	98.15	94.48	49.07	0.0247	79.87
Experimental group (34°C)							
GM1	47,388,262	45,363,630	98.21	94.62	50.43	0.0246	78.07
GM2	46,657,000	45,108,462	97.92	93.88	48.95	0.0253	78.64
GM3	48,723,702	46,616,836	97.95	93.97	48.4	0.0252	79.05

GC1–GC3 are the three parallel experimental group samples of the control group. GM1–GM3 are the three parallel experimental group samples of the heat stress group.

### 3.3 Identification of DEGs

**FIGURE 1:**
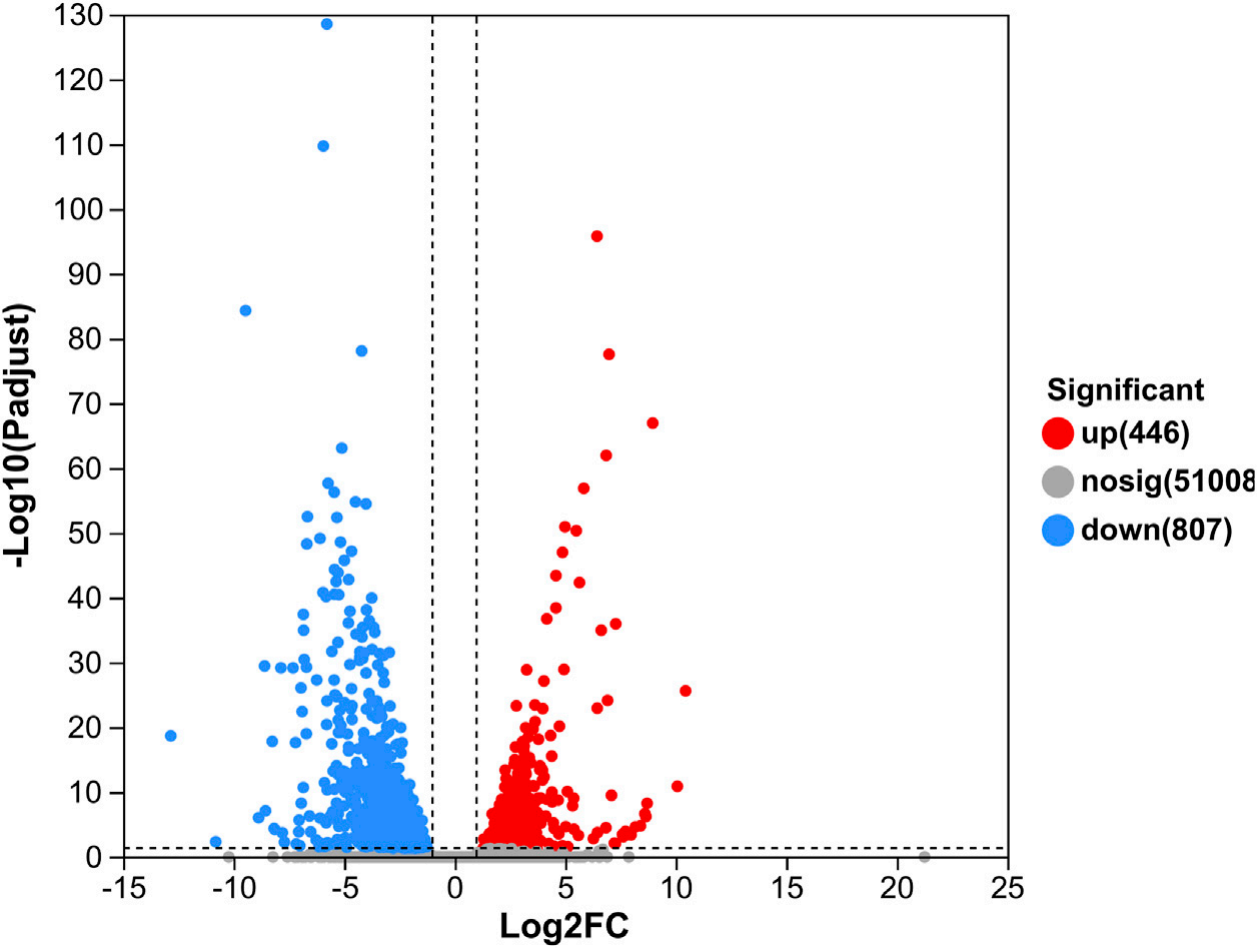
Volcano plot of DEGs in GM vs. GC groups. Each dot represents one gene, red color indicates significantly upregulated genes, blue color indicates significantly downregulated genes, and gray color indicates non-significantly differentially expressed genes.

**FIGURE 2:**
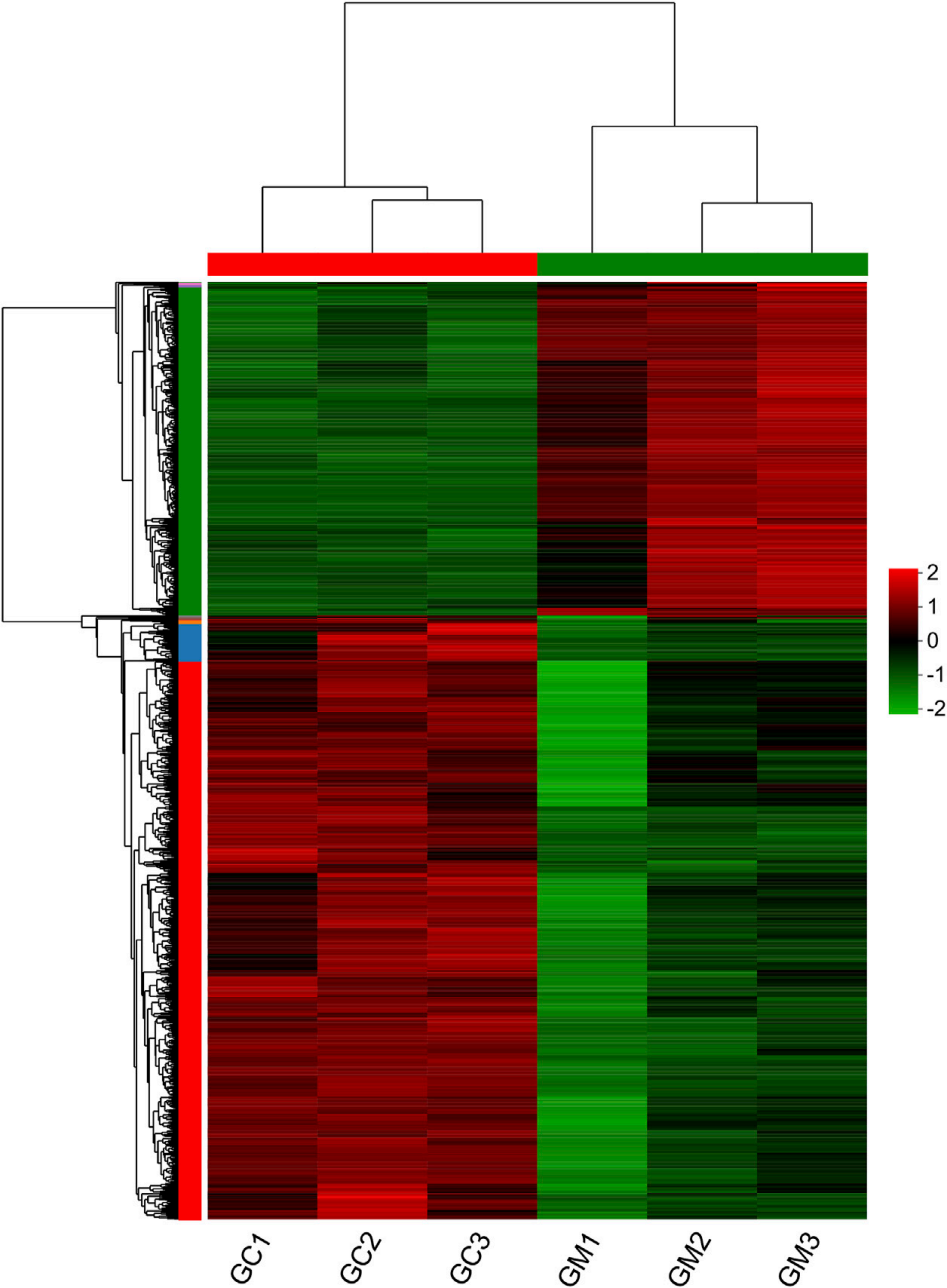
Heatmap of DEGs in GM vs. GC groups. Each row represents one gene. Colors ranging from green to red indicate gene expression from low to high.

In our study, 1,253 DEGs were identified, including 446 upregulated genes and 807 downregulated genes (Figure 1). Clustering analysis indicated that the GC and GM groups were clustered separately, with quite different expression patterns in each group (Figure 2).

### 3.4 Enrichment analysis

**FIGURE 3:**
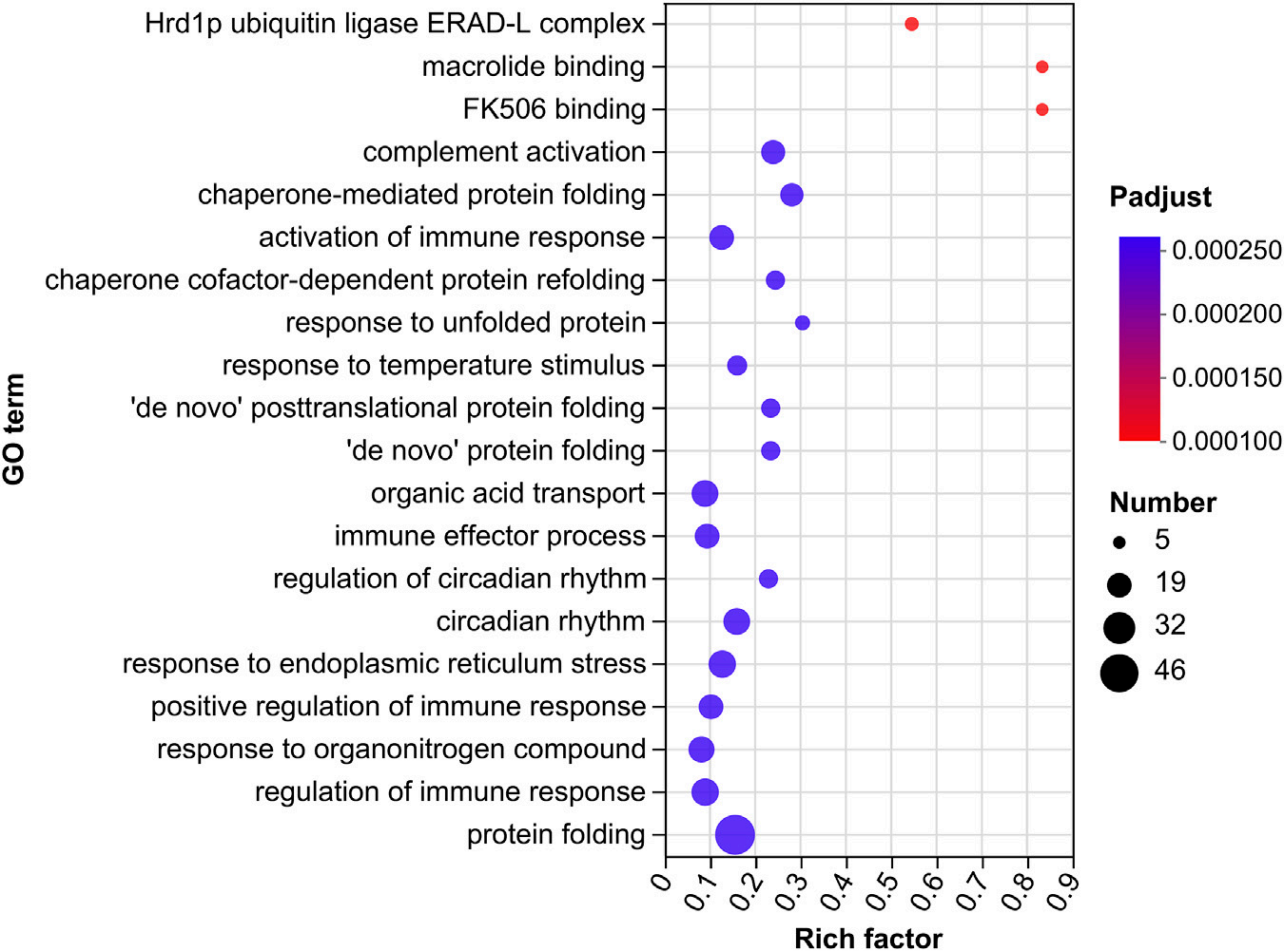
GO enrichment analysis of DEGs in GM vs. GC groups. Colors ranging from blue to red indicate GO term significance from low to high. The size of the dot represents the number of genes enriched from more to less.

Gene Ontology (GO) terms are classified into three basic categories: biological processes (BPs), cellular components (CCs), and molecular functions (MFs). There were 255 GO terms significantly enriched in the GM vs. GC groups (*p* < 0.05), which contain 144 BPs, 73 MFs and 38 CCs (Figure 3; Supplementary Table S2).

**FIGURE 4:**
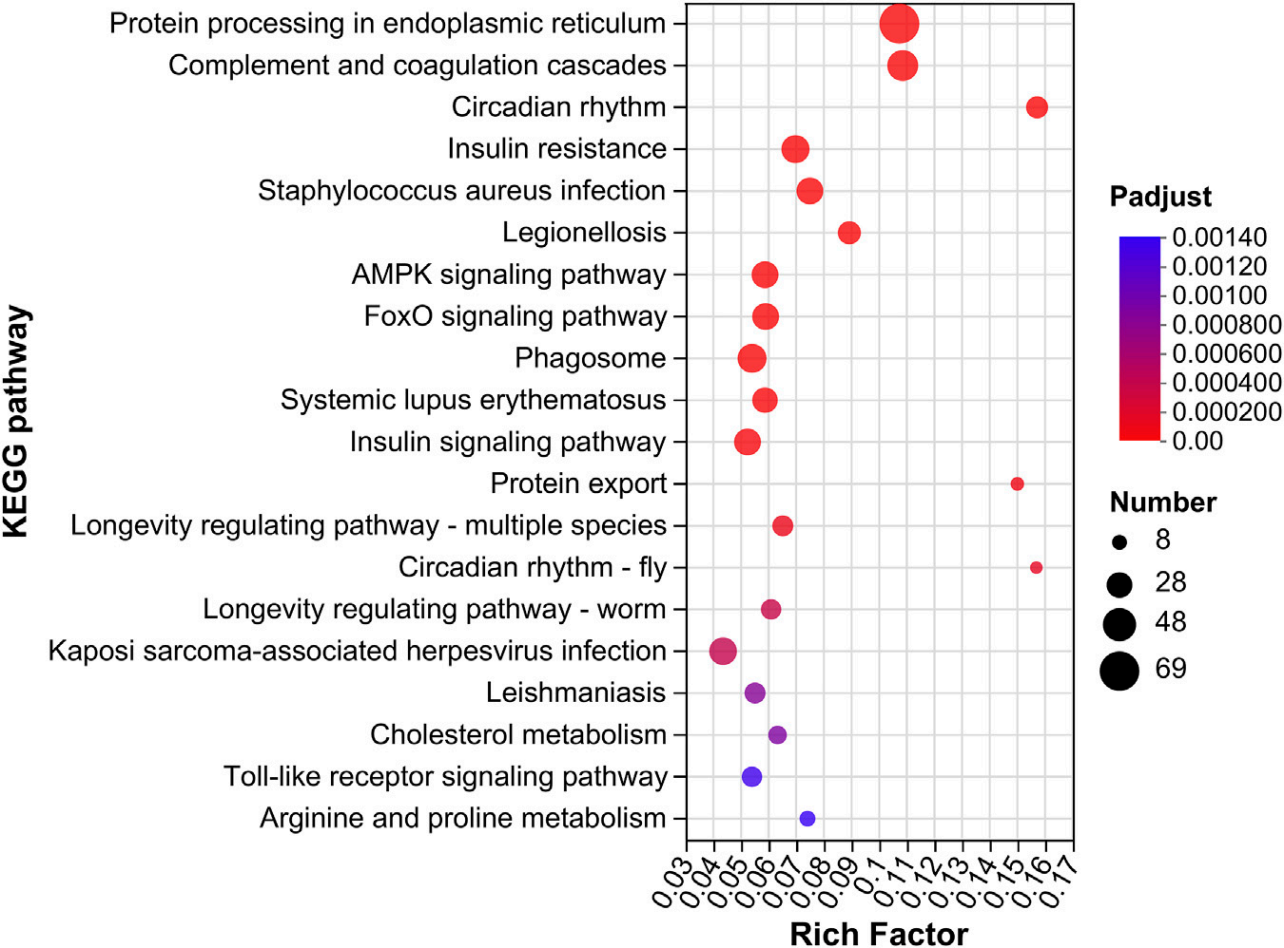
KEGG enrichment analysis of DEGs in GM vs. GC groups. Colors ranging from blue to red indicate the KEGG pathway significance from low to high. The size of the dot represents the number of genes enriched from more to less.

In the GM vs. GC groups, we found 57 KEGG pathways that were significantly enriched (*p* < 0.05). Within these pathways, “protein processing in endoplasmic reticulum, map04141” was the most significantly enriched pathway. Other pathways such as “AMPK signaling pathway, map04152,” “FoxO signaling pathway, map04068,” and “protein export, map03060” were also significantly enriched (Figure 4; Supplementary Table S3).

**FIGURE 5:**
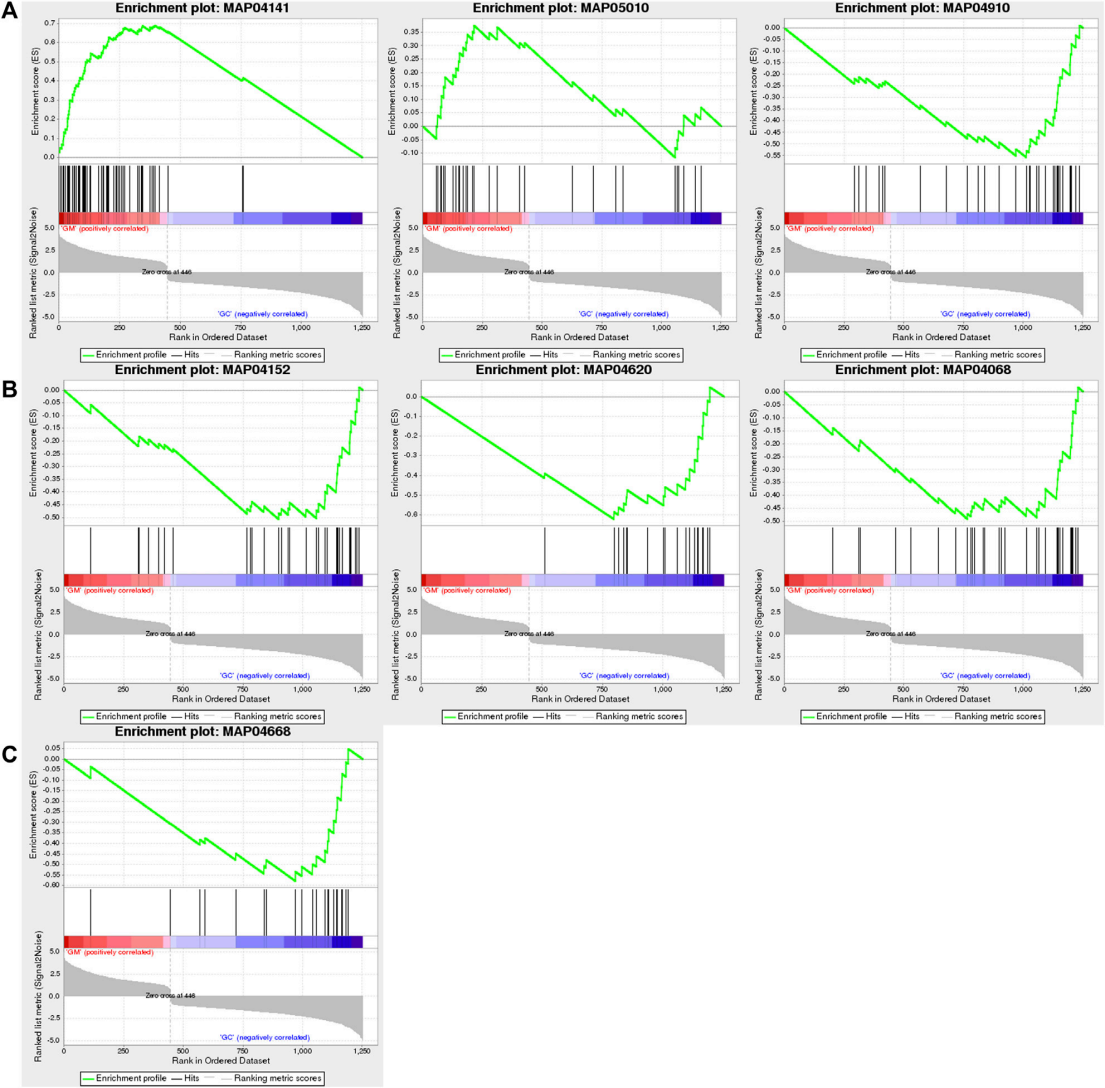
GSEA of DEGs in GM vs. GC groups. **(A)** “Protein processing in endoplasmic reticulum,” “Alzheimer’s disease,” and “insulin signaling pathway” are shown from left to right. **(B)** “AMPK signaling pathway,” “Toll-like receptor signaling pathway,” and “FoxO signaling pathway” are shown from left to right. **(C)** “TNF signaling pathway.”

Through GSEA, we also found that only “protein processing in endoplasmic reticulum (NES = 4.52)” and “Alzheimer’s disease (NES = 1.93)” were positively enriched, while “insulin signaling pathway (NES = −2.62),” “AMPK signaling pathway (NES = −2.43),” “Toll-like receptor signaling pathway (NES = −2.42),” “TNF signaling pathway (NES = −2.37),” and “FoxO signaling pathway (NES = −2.29)” were negatively enriched (Figure 5; Supplementary Table S4).

### 3.5 Verification of RNA-seq using RT-qPCR

**FIGURE 6:**
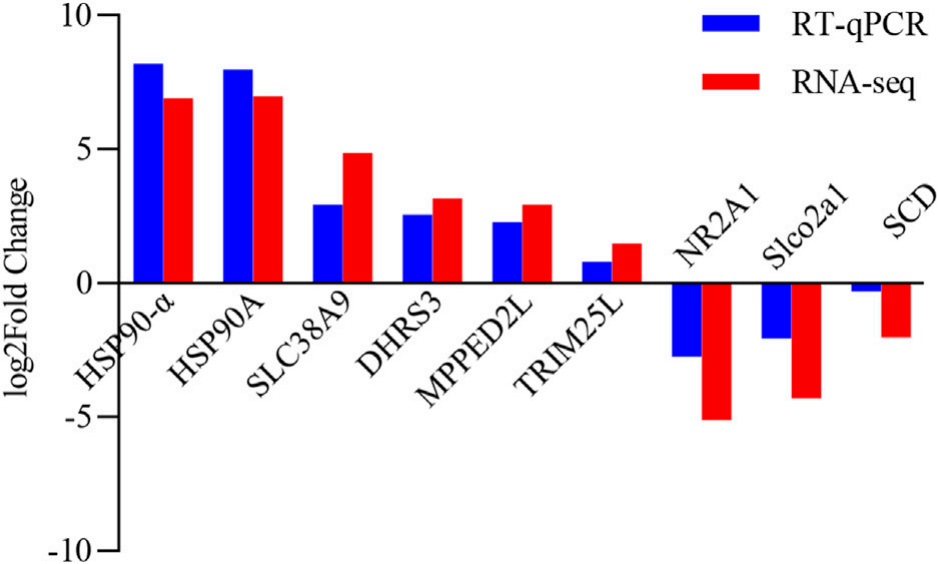
Comparison of gene expression between RT-qPCR and RNA-seq, with gene names on the *X*-axis and log_2_ value of relative gene expression on the *Y*-axis.

In order to determine the accuracy and reliability of RNA-seq, nine genes were randomly chosen for RT-qPCR validation. In both RNA-seq and RT-qPCR, the chosen genes had consistent expression patterns (Figure 6).

## 4 Discussion

Domestication is a widely known example of artificial selection and has helped understand some of the most extreme within-species phenotypic variations over the years (Hoglund et al., 2020). PF-carp has been domesticated by humans for more than 1,200 years in paddy fields, developing phenotypic traits and genetic structure to adapt to the environment of the paddy fields (Ren et al., 2018; Qi et al., 2020). The liver of fish plays a crucial role in metabolic and immunological processes (Nakamura and Nishina, 2009). Many studies have reported the effect of heat stress on the structure and function of the liver (Li B et al., 2019; Dettleff et al., 2022; Yang et al., 2022). However, underlying physiological and molecular regulatory mechanisms of hepatopancreases in PF-carp responses to the high-temperature environment of shallow paddy fields remain elusive. Therefore, to better understand the adaptation mechanism of PF-carp to the paddy field environment after extensive domestication, we assessed the pertinent physiological parameters and performed RNA-seq on PF-carp that were under heat stress.

In our current study, only SOD activity and TG content showed significant changes in serum, and MDA content in the hepatopancreas also showed significant changes after undergoing 24 h of high-temperature stress at 34°C, whereas the rest of the various enzyme activity indexes measured in serum and hepatopancreas did not show any significant changes. Fish have been reported to produce large amounts of ROS at high temperatures (Messina et al., 2023). In fish, SOD is the most important system that is used as the first line of defense against oxidative stress to remove the ROS that is released as a response to heat stress (Wang et al., 2019). Free radicals attack unsaturated fatty acids in the cell membrane to produce MDA (Papadimitriou and Loumbourdis, 2002). However, only SOD activity in the serum antioxidant defense system showed significantly changes, and MDA content in the hepatopancreas was significantly upregulated after 24 h of heat stress, suggesting that the body of PF-carp does not undergo stress when experiencing heat stress in rice paddy fields. In contrast, the antioxidant systems of *Acipenser baerii* (*A. baerii*) (Yang et al., 2021), rainbow trout (*Oncorhynchus mykiss*) (Li et al., 2022), and pikeperch (*Sander lucioperca*) (Li, C. et al., 2019) are significantly activated after thermal stress to maintain homeostasis in their internal environment.

TG is the main form of intracellular fat in fish and is critical for the storage and supply of energy (Komprda et al., 2014). GLU is the primary source of energy in fish that is produced by the digestion and absorption of glucose from meals and the breakdown of glycogen in the liver (Mommsen et al., 1999). TG levels increased significantly during heat stress in PF-carp, while the GLU content did not significantly change, indicating that PF-carp does not need to consume energy during heat stress. Certain particular enzymes may be released into the blood by damaged tissues or organs (Islam et al., 2021). It is reported that cytolysis and enzyme leakage into the bloodstream could lead to an increase in ALT and AST levels, which suggests liver damage (Bacchetta et al., 2014). In our findings, serum levels of ALT and AST in PF-carp during heat stress showed no significant changes, suggesting that heat stress did not affect the dysfunction of the hepatopancreas. In addition, Japanese flounder (*Paralichthys olivaceus*) (Han et al., 2023), pikeperch (*Sander lucioperca*) (Chen, Y. et al., 2021), and largemouth bass (*Micropterus salmoides*) (Zhao et al., 2022) often show damage to their tissues after experiencing heat stress.

RNA-seq has been well established and used to study the impact of high temperatures on fish, such as channel catfish (*Ictalurus punctatus*) (Tan et al., 2019), turbot (*Scophthalmus maximus*) (Huang, Z. et al., 2022), and blunt snout bream (*Megalobrama amblycephala*) (Li, B. et al., 2019). In this novel study, RNA-seq was used to determine the molecular changes in hepatopancreases of PF-carp. Through GO enrichment analysis, we reported that protein processing was significantly enriched. Furthermore, protein processing in the endoplasmic reticulum was the most significantly enriched pathway identified via KEGG enrichment analysis. According to the GSEA results, it was demonstrated that protein processing in the endoplasmic reticulum was the most significantly enriched and upregulated gene set. The endoplasmic reticulum is essential for intracellular calcium homeostasis, modification, and transport, as well as protein synthesis and folding (Kang and Jeon, 2021). The change in temperature could cause ER stress and protein misfolding (Tang et al., 2023). Heat shock proteins (HSPs) aid the folding and function of many proteins and could prevent protein misfolding (Lanneau et al., 2010). In addition, misfolded proteins through endoplasmic reticulum-associated protein degradation (ERAD) can be promoted by the coordination between the HSPs and ubiquitin ligase (Bozaykut et al., 2014; Kang and Jeon, 2021). In our study, HSP40, HSP70, HSP90, and various genes related to ubiquitin ligase were significantly upregulated. Therefore, we hypothesized that PF-carp removes abnormal proteins from the body mainly through protein processing in the endoplasmic reticulum during acclimatization to the high-temperature environment of shallow paddy fields. This is consistent with the way in which Atlantic salmon (*Salmo salar*) (Shi et al., 2019), rainbow trout (*Oncorhynchus mykiss*) (Li et al., 2017), and largemouth bass (*Micropterus salmoides*) (Zhao et al., 2022) maintain cellular homeostasis during heat stress.

Furthermore, GSEA revealed that the insulin, AMPK, FoxO , Toll-like receptor, and TNF signaling pathways were downregulated and insulin, AMPK, and FoxO signaling pathways could regulate body energy metabolism (Glauser and Schlegel, 2007; Wang et al., 2015; Schell et al., 2021). The insulin signaling pathways could be activated and take part in regulating glucose production in the liver. Additionally, it plays a role in the absorption of glucose into fat and muscle cells, helping maintain the body’s glucose balance. (Suren Garg et al., 2023). A variety of physiological stimuli could activate the AMPK signaling pathway, such as glucose deprivation and oxidative stress, which may lead to a reduction in the cellular energy level and an increase in the AMP/ATP ratio (Schultze et al., 2012). Many genes in the FoxO signaling pathway are involved in the production of fat and glucose, and their upregulated expression can stimulate the production of these substances (Gross et al., 2009). It is reported that when fish are subjected to stress, the organism spends a large amount of energy to protect itself from external stresses (Petitjean et al., 2019). For example, gilthead sea bream (*Sparus aurata L.*) will improve mitochondrial metabolism when it faces stress (Bermejo-Nogales et al., 2014). However, these signaling pathways associated with metabolism were downregulated in our results, suggesting that PF-carp does not need to consume a lot of energy to adapt to the high-temperature environment of shallow paddy fields, which is also consistent with the results of our physiological parameters. Therefore, we believe that it is well adapted to paddy fields.

When the organism is exposed to heat stress, it activates a variety of immunomodulatory pathways, such as the Toll-like receptor and TNF signaling pathways. The activation of these immune pathways mediates the inflammatory response and reduces the damage caused by heat stress (Basu et al., 2015; Huang, T. et al., 2022). However, these immune pathways were downregulated when PF-carp was subjected to heat stress, suggesting that the organism did not initiate immune regulation. Pikeperch (*Sander lucioperca*) (Liu et al., 2022), rainbow trout (*Oncorhynchus mykiss*) (Guo et al., 2023), and grass carp (*Ctenopharyngodon idella*) (Zhang et al., 2022) activate these immune pathways to fight against damage when exposed to heat stress.

In a word, to maintain cellular homeostasis in PF-carp, protein processing in the endoplasmic reticulum plays a key role when it is subjected to high temperature stress in shallow paddy fields. In addition, the organism does not produce a stress response, nor does it consume a large amount of energy and trigger an inflammatory response to withstand any harm caused by heat stress. Instead, it adapts well to the high-temperature environment of the paddy field.

## Data Availability

The datasets presented in this study can be found in online repositories. The names of the repository/repositories and accession number(s) can be found in the article/Supplementary Material.

## References

[B1] Bacchetta C. Rossi A. Ale A. Campana M. Julieta Parma M. Cazenave J. (2014). Combined toxicological effects of pesticides: a fish multi-biomarker approach. Ecol. Indic. 36, 532–538. 10.1016/j.ecolind.2013.09.016

[B2] Balon E. K. (2004). About the oldest domesticates among fishes. J. Fish Biol. 65 (1), 1–27. 10.1111/j.0022-1112.2004.00563.x

[B3] Basu M. Paichha M. Swain B. Lenka S. S. Singh S. Chakrabarti R. (2015). Modulation of TLR2, TLR4, TLR5, NOD1 and NOD2 receptor gene expressions and their downstream signaling molecules following thermal stress in the Indian major carp catla (Catla catla). 3 Biotech. 5 (6), 1021–1030. 10.1007/s13205-015-0306-5 PMC4624144 28324409

[B4] Bermejo-Nogales A. Nederlof M. Benedito-Palos L. Ballester-Lozano G. F. Folkedal O. Olsen R. E. (2014). Metabolic and transcriptional responses of gilthead sea bream (*Sparus aurata* L.) to environmental stress: new insights in fish mitochondrial phenotyping. Gen. Comp. Endocrinol. 205, 305–315. 10.1016/j.ygcen.2014.04.016 24792819

[B5] Bozaykut P. Ozer N. K. Karademir B. (2014). Regulation of protein turnover by heat shock proteins. Free Radic. Biol. Med. 77, 195–209. 10.1016/j.freeradbiomed.2014.08.012 25236750

[B6] Chen X X. Tang J. Hu L. Wu M. Ren W. (2021). Rice-fish system in qingtian: Ecology, conservation & utilization. The science press of China.

[B7] Chen Y Y. Liu E. Li C. Pan C. Zhao X. Wang Y. (2021). Effects of heat stress on histopathology, antioxidant enzymes, and transcriptomic profiles in gills of pikeperch *Sander lucioperca* . Aquaculture 534. 10.1016/j.aquaculture.2020.736277

[B8] Dettleff P. Zuloaga R. Fuentes M. Gonzalez P. Aedo J. Manuel Estrada J. (2022). High-temperature stress effect on the red cusk-eel (geypterus chilensis) liver: transcriptional modulation and oxidative stress damage. Biology-Basel 11 (7), 990. 10.3390/biology11070990 36101373 PMC9312335

[B9] Diamond J. (2002). Evolution, consequences and future of plant and animal domestication. Nature 418 (6898), 700–707. 10.1038/nature01019 12167878

[B10] Glauser D. A. Schlegel W. (2007). The emerging role of FOXO transcription factors in pancreatic beta cells. J. Endocrinol. 193 (2), 195–207. 10.1677/JOE-06-0191 17470511

[B11] Gross D. N. Wan M. Birnbaum M. J. (2009). The role of FOXO in the regulation of metabolism. Curr. Diabetes Rep. 9 (3), 208–214. 10.1007/s11892-009-0034-5 19490822

[B12] Guo H. Whitehouse L. Danzmann R. Dixon B. (2023). Effects of juvenile thermal preconditioning on the heat-shock, immune, and stress responses of rainbow trout upon a secondary thermal challenge. Comp. Biochem. Physiol. A Mol. Integr. Physiol. 280, 111413. 10.1016/j.cbpa.2023.111413 36893937

[B13] Han P. Qiao Y. He J. Wang X. (2023). Stress responses to warming in Japanese flounder (*Paralichthys olivaceus*) from different environmental scenarios. Sci. Total Environ. 897, 165341. 10.1016/j.scitotenv.2023.165341 37414161

[B14] Hoglund A. Henriksen R. Fogelholm J. Churcher A. M. Guerrero-Bosagna C. M. Martinez-Barrio A. (2020). The methylation landscape and its role in domestication and gene regulation in the chicken. Nat. Ecol. Evol. 4 (12), 1713–1724. 10.1038/s41559-020-01310-1 32958860 PMC7616959

[B15] Huang T T. Gu W. Liu E. Wang B. Wang G. Dong F. (2022). miR-301b-5p and its target gene nfatc2ip regulate inflammatory responses in the liver of rainbow trout (*Oncorhynchus mykiss*) under high temperature stress. Ecotoxicol. Environ. Saf. 242, 113915. 10.1016/j.ecoenv.2022.113915 35901591

[B16] Huang Z Z. Guo X. Wang Q. Ma A. Zhao T. Qiao X. (2022). Digital RNA-seq analysis of the cardiac transcriptome response to thermal stress in turbot *Scophthalmus maximus* . J. Therm. Biol. 104, 103141. 10.1016/j.jtherbio.2021.103141 35180952

[B17] Islam M. J. Kunzmann A. Slater M. J. (2021). Extreme winter cold-induced osmoregulatory, metabolic, and physiological responses in European seabass (*Dicentrarchus labrax*) acclimatized at different salinities. Sci. Total Environ. 771, 145202. 10.1016/j.scitotenv.2021.145202 33736134

[B18] Kang J. A. Jeon Y. J. (2021). How is the fidelity of proteins ensured in terms of both quality and quantity at the endoplasmic reticulum? Mechanistic insights into E3 ubiquitin ligases. Int. J. Mol. Sci. 22 (4), 2078. 10.3390/ijms22042078 33669844 PMC7923238

[B19] Komprda T. Zornikova G. Knoll A. Vykoukalova Z. Rozikova V. Skultety O. (2014). Effect of dietary eicosapentaenoic and docosahexaenoic acid on expression of rat liver genes controlling cholesterol homeostasis and on plasma cholesterol level. Czech J. Animal Sci. 59 (9), 391–398. 10.17221/7650-cjas

[B20] Lanneau D. Wettstein G. Bonniaud P. Garrido C. (2010). Heat shock proteins: cell protection through protein triage. Thescientificworldjournal 10, 1543–1552. 10.1100/tsw.2010.152 20694452 PMC5763791

[B21] Li B B. Sun S. Zhu J. Yanli S. Wuxiao Z. Ge X. (2019). Transcriptome profiling and histology changes in juvenile blunt snout bream (*Megalobrama amblycephala*) liver tissue in response to acute thermal stress. Genomics 111 (3), 242–250. 10.1016/j.ygeno.2018.11.011 30458273

[B22] Li C C. Wang Y. Wang G. Chen Y. Guo J. Pan C. (2019). Physicochemical changes in liver and Hsc70 expression in pikeperch *Sander lucioperca* under heat stress. Ecotoxicol. Environ. Saf. 181, 130–137. 10.1016/j.ecoenv.2019.05.083 31176247

[B23] Li Y. J. Huang J. Q. Liu Z. Zhou Y. J. Xia B. P. Wang Y. J. (2017). Transcriptome analysis provides insights into hepatic responses to moderate heat stress in the rainbow trout (*Oncorhynchus mykiss*). Gene 619, 1–9. 10.1016/j.gene.2017.03.041 28365313

[B24] Li S. Liu Y. Li B. Ding L. Wei X. Wang P. (2022). Physiological responses to heat stress in the liver of rainbow trout (*Oncorhynchus mykiss*) revealed by UPLC-QTOF-MS metabolomics and biochemical assays. Ecotoxicol. Environ. Saf. 242, 113949. 10.1016/j.ecoenv.2022.113949 35999764

[B25] Liu E. Zhao X. Li C. Wang Y. Li L. Zhu H. (2022). Effects of acute heat stress on liver damage, apoptosis and inflammation of pikeperch (*Sander lucioperca*). J. Therm. Biol. 106, 103251. 10.1016/j.jtherbio.2022.103251 35636889

[B26] Lu J. Li X. (2006). Review of rice-fish-farming systems in China - one of the globally important ingenious agricultural heritage systems (GIAHS). Aquaculture 260 (1-4), 106–113. 10.1016/j.aquaculture.2006.05.059

[B27] Messina S. Costantini D. Eens M. (2023). Impacts of rising temperatures and water acidification on the oxidative status and immune system of aquatic ectothermic vertebrates: a meta-analysis. Sci. Total Environ. 868, 161580. 10.1016/j.scitotenv.2023.161580 36646226

[B28] Mommsen T. P. Vijayan M. M. Moon T. W. (1999). Cortisol in teleosts: dynamics, mechanisms of action, and metabolic regulation. Rev. Fish Biol. Fish. 9 (3), 211–268. 10.1023/a:1008924418720

[B29] Nakamura T. Nishina H. (2009). Liver development: lessons from knockout mice and mutant fish. Hepatology Res. 39 (7), 633–644. 10.1111/j.1872-034X.2009.00522.x 19456896

[B30] Papadimitriou E. Loumbourdis N. S. (2002). Exposure of the frog *Rana ridibunda* to copper: impact on two biomarkers, lipid peroxidation, and glutathione. Bull. Environ. Contam. Toxicol. 69 (6), 885–891. 10.1007/s00128-002-0142-2 12428167

[B31] Petitjean Q. Jean S. Gandar A. Cote J. Laffaille P. Jacquin L. (2019). Stress responses in fish: from molecular to evolutionary processes. Sci. Total Environ. 684, 371–380. 10.1016/j.scitotenv.2019.05.357 31154210

[B32] Qi M. Wu Q. Liu T. Hou Y. Miao Y. Hu M. (2020). Hepatopancreas transcriptome profiling analysis reveals physiological responses to acute hypoxia and reoxygenation in juvenile qingtian paddy field carp *Cyprinus carpio* var qingtianensis. Front. Physiol. 11, 1110. 10.3389/fphys.2020.01110 33041847 PMC7518031

[B33] Ren W. Hu L. Guo L. Zhang J. Tang L. Zhang E. (2018). Preservation of the genetic diversity of a local common carp in the agricultural heritage rice-fish system. Proc. Natl. Acad. Sci. U. S. A. 115 (3), E546–E554. 10.1073/pnas.1709582115 29295926 PMC5776965

[B34] Schell M. Wardelmann K. Kleinridders A. (2021). Untangling the effect of insulin action on brain mitochondria and metabolism. J. Neuroendocrinol. 33 (4), e12932. 10.1111/jne.12932 33506556

[B35] Schultze S. M. Hemmings B. A. Niessen M. Tschopp O. (2012). PI3K/AKT, MAPK and AMPK signalling: protein kinases in glucose homeostasis. Expert Rev. Mol. Med. 14, e1. 10.1017/S1462399411002109 22233681

[B36] Shi K. P. Dong S. L. Zhou Y. G. Li Y. Gao Q. F. Sun D. J. (2019). RNA-seq reveals temporal differences in the transcriptome response to acute heat stress in the Atlantic salmon (*Salmo salar*). Comp. Biochem. Physiology D-Genomics Proteomics 30, 169–178. 10.1016/j.cbd.2018.12.011 30861459

[B37] Suren Garg S. Kushwaha K. Dubey R. Gupta J. (2023). Association between obesity, inflammation and insulin resistance: insights into signaling pathways and therapeutic interventions. Diabetes Res. Clin. Pract. 200, 110691. 10.1016/j.diabres.2023.110691 37150407

[B38] Tan S. Wang W. Tian C. Niu D. Zhou T. Jin Y. (2019). Heat stress induced alternative splicing in catfish as determined by transcriptome analysis. Comp. Biochem. Physiol. Part D. Genomics Proteomics 29, 166–172. 10.1016/j.cbd.2018.11.008 30481682

[B39] Tang Z. Yang Y. Wu Z. Ji Y. (2023). Heat stress-induced intestinal barrier impairment: current insights into the aspects of oxidative stress and endoplasmic reticulum stress. J. Agric. Food Chem. 71 (14), 5438–5449. 10.1021/acs.jafc.3c00798 37012901

[B40] Wang B. Liu Y. Feng L. Jiang W. D. Kuang S. Y. Jiang J. (2015). Effects of dietary arginine supplementation on growth performance, flesh quality, muscle antioxidant capacity and antioxidant-related signalling molecule expression in young grass carp (Ctenopharyngodon idella). Food Chem. 167, 91–99. 10.1016/j.foodchem.2014.06.091 25148964

[B41] Wang Y. Li C. Pan C. Liu E. Zhao X. Ling Q. (2019). Alterations to transcriptomic profile, histopathology, and oxidative stress in liver of pikeperch (*Sander lucioperca*) under heat stress. Fish Shellfish Immunol. 95, 659–669. 10.1016/j.fsi.2019.11.014 31706008

[B42] Xie J. Hu L. Tang J. Wu X. Li N. Yuan Y. (2011). Ecological mechanisms underlying the sustainability of the agricultural heritage rice-fish coculture system. Proc. Natl. Acad. Sci. U. S. A. 108 (50), E1381–E1387. 10.1073/pnas.1111043108 22084110 PMC3250190

[B43] Xu P. Zhang X. Wang X. Li J. Liu G. Kuang Y. (2014). Genome sequence and genetic diversity of the common carp, *Cyprinus carpio* . Nat. Genet. 46 (11), 1212–1219. 10.1038/ng.3098 25240282

[B44] Yang S. Yang X. Li Y. Li D. Gong Q. Huang X. (2021). The multilevel responses of *Acipenser baerii* and its hybrids (*A. baerii* ♀ × A. schrenckii ♂) to chronic heat stress. Aquaculture 541, 736773. 10.1016/j.aquaculture.2021.736773

[B45] Yang C. Dong J. Sun C. Li W. Tian Y. Liu Z. (2022). Exposure to heat stress causes downregulation of immune response genes and weakens the disease resistance of *Micropterus salmoides* . Comp. Biochem. Physiology D-Genomics Proteomics 43, 101011. 10.1016/j.cbd.2022.101011 35839613

[B46] Zhang W. Xu X. Li J. Shen Y. (2022). Transcriptomic analysis of the liver and brain in grass carp (Ctenopharyngodon idella) under heat stress. Mar. Biotechnol. (NY) 24 (5), 856–870. 10.1007/s10126-022-10148-6 35930066

[B47] Zhao X. Li L. Li C. Liu E. Zhu H. Ling Q. (2022). Heat stress-induced endoplasmic reticulum stress promotes liver apoptosis in largemouth bass (*Micropterus salmoides*). Aquaculture 546, 737401. 10.1016/j.aquaculture.2021.737401

